# Supplementary material for: Identification of photoperiod-induced specific miRNAs in the adrenal glands of Sunite sheep (Ovis aries)
Source: Front Vet Sci. 2022 Jul 22;9:888207. doi: 10.3389/fvets.2022.888207 (PMC9354845; doi:10.3389/fvets.2022.888207)
Supplement: Supplementary file 1 [file Data_Sheet_1.ZIP › Supplementary file/miRDeep2 file.zip!/miRDeep2 file/Know_Structure/SP42A1/oar-mir-433.pdf]

miRBase precursor : oar-mir-433
Total read count : 1002
oar-miR-433-5p read count : 1
oar-miR-433-3p read count : 1001
remaining reads : 0

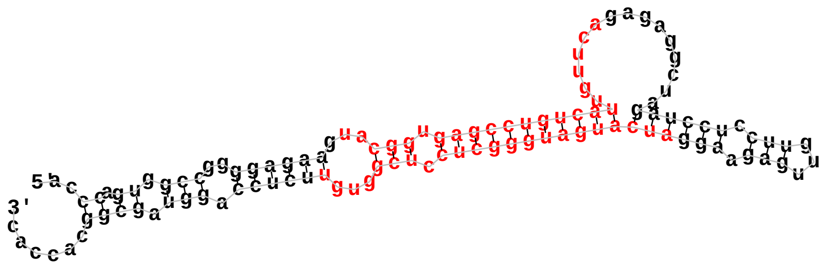

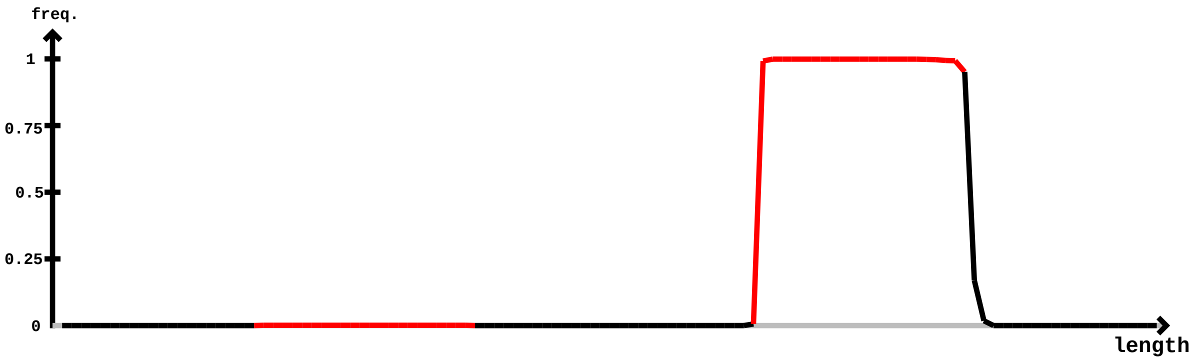


```
                    oar-miR-433-5p
                                                                        oar-miR-433-3p
5'- acccaguggccggggagaaguacggugagccugucauuguucagagaggcuagauccuccuuguugagaaggaucaugaugggcuccucggugu ucuccagguagcggcaccac   -3'    exp
    ..((.((.(((..((((((...(((.(((((((((((...............((((((.(((...))).)))))))))))))))))).)))...)))))).))).))))......   reads   mm   sample
    ....................uacggugagccugucauuguuc........................................................................   1       0    seq
    .......................................................................gaucaugaugggcuccucggug.....................   1       0    seq
    .......................................................................gaucaugaugggcuccucggugu....................   5       0    seq
    ........................................................................aucaugaugggcuccuc.........................   1       0    seq
    ........................................................................aucaugaugggcuccucg........................   1       0    seq
    ........................................................................aucaugaugggcuccucgg.......................   3       0    seq
    ........................................................................aucaugaugggcuccucggu......................   1       0    seq
    ........................................................................aucaugaugggcuccucggug.....................   41      0    seq
    ........................................................................aucaugaugggcuccucggugu....................   772     0    seq
    ........................................................................aucaugaugggcuccucgguguu...................   151     0    seq
    ........................................................................aucaugaugggcuccucgguguuc..................   18      0    seq
    .........................................................................ucaugaugggcuccucggugu....................   6       0    seq
    .........................................................................ucaugaugggcuccucgguguu...................   1       0    seq
```
